# Characterization and transferability of microsatellite markers of the cultivated peanut (*Arachis hypogaea*)

**DOI:** 10.1186/1471-2229-7-9

**Published:** 2007-02-27

**Authors:** Marcos A Gimenes, Andrea A Hoshino, Andrea VG Barbosa, Dario A Palmieri, Catalina R Lopes

**Affiliations:** 1 Laboratório de Biotecnologia e Genética Molecular (BIOGEM), Departamento de Genética, Instituto de Biociências, Universidade Estadual Paulista (UNESP), Botucatu, SP, Brazil; 2 Instituto Agronômico de Campinas – RGV, Caixa Postal 28, Campinas, SP, Brazil

## Abstract

**Background:**

The genus *Arachis *includes *Arachis hypogaea *(cultivated peanut) and wild species that are used in peanut breeding or as forage. Molecular markers have been employed in several studies of this genus, but microsatellite markers have only been used in few investigations. Microsatellites are very informative and are useful to assess genetic variability, analyze mating systems and in genetic mapping. The objectives of this study were to develop *A. hypogaea *microsatellite loci and to evaluate the transferability of these markers to other *Arachis *species.

**Results:**

Thirteen loci were isolated and characterized using 16 accessions of *A. hypogaea*. The level of variation found in *A. hypogaea *using microsatellites was higher than with other markers. Cross-transferability of the markers was also high. Sequencing of the fragments amplified using the primer pair Ah11 from 17 wild *Arachis *species showed that almost all wild species had similar repeated sequence to the one observed in *A. hypogaea*. Sequence data suggested that there is no correlation between taxonomic relationship of a wild species to *A. hypogaea *and the number of repeats found in its microsatellite loci.

**Conclusion:**

These results show that microsatellite primer pairs from *A. hypogaea *have multiple uses. A higher level of variation among *A. hypogaea *accessions can be detected using microsatellite markers in comparison to other markers, such as RFLP, RAPD and AFLP. The microsatellite primers of *A. hypogaea *showed a very high rate of transferability to other species of the genus. These primer pairs provide important tools to evaluate the genetic variability and to assess the mating system in *Arachis *species.

## Background

The origin and the diversity center of the genus *Arachis *are in South America [1]. This genus comprises 69 species, most of which are diploid and wild. The cultivated species include *A. hypogaea *L., the cultivated peanut, *A. glabrata *and *A. pintoi*, which have been used in forage production [2,3]. This genus is divided into nine sections (*Arachis*, *Erectoides*, *Heteranthae*, *Caulorrhizae*, *Rhizomatosae*, *Extranervosae*, *Triseminatae*, *Procumbentes *and *Trierectoides*) according to their morphology, geographic distribution and sexual compatibility [1].

The extensive morphological variation in *A. hypogaea *has led to the identification of subspecies, although studies using molecular markers have found little polymorphism in the germplasm of this species [4-6]. The observed restriction in genetic variation limits the use of several approaches, such as molecular marker-assisted selection and the construction of a molecular map that are essential tools in *A. hypogaea *breeding.

Cultivated peanut is an allotetraploid that contains genomes A and B, which are found in wild diploid species of section *Arachis*. This species has arisen probably from a unique cross between the wild diploid species *A. duranensis *(A genome) and *A. ipaënsis *(B genome) resulting in a hybrid whose chromosome number was spontaneously duplicated [7]. This duplication isolated *A. hypogaea *from the wild diploid species not allowing allele exchange with them. The origin through a single and recent polyplodization event, followed by successive selection during breeding efforts, resulted in a highly conserved genome [8]. The morphological variation observed among accessions of *A. hypogaea *is most probably due to the variation in few genes [9].

Microsatellites are highly polymorphic molecular markers [10], which have been used to analyze genetic variability and to construct molecular maps in several plant species [11-14]. Hopkins and colleagues [15] analyzed the genetic variation using six microsatellite primer pairs and 19 accessions of *A. hypogaea *and three accessions of wild *Arachis *species (*A. duranensis*, *A. ipaënsis*, *A. monticola*). These authors have observed that despite the low frequency of polymorphism found in *A. hypogaea*, these microsatellite loci were very informative and could provide a useful tool to identify and partition genetic variation in the cultivated peanut. Fergurson and colleagues [16] developed 226 microsatellite primer pairs for *A. hypogaea *and from the 192 that amplified well 110 putative loci showed polymorphism in a diverse array of 24 cultivated peanut accessions. Moretzsohn and colleagues [17] analyzing 36 species of *Arachis *observed the cross species amplification rate of *A. hypogaea *microsatellite primers was up 76% to species of section *Arachis *and up to 45% to species of the other eight section of genus *Arachis*.

Microsatellite markers could be useful to analyze the genetic variation in the germplasm of wild *Arachis *species. These species have more intraspecific genetic variation detectable than *A. hypogaea*, as shown by using molecular markers [18,19], and are resistant to numerous pests and diseases that affect the cultivated peanut [20]. The high cost of developing microsatellite markers is the main factor limiting their widespread use in this genus. A good alternative would be the use of a set of primers to obtain cross-species transferability, as reported in other studies [21-24].

The objectives of this study were to isolate and characterize the microsatellite loci of *A. hypogaea *and to assess the cross-transferability of these markers to other *Arachis *species.

## Results and Discussion

A total of 68 random clones were selected and sequenced. Thirty-eight (55.9%) of them contained microsatellites. Repeat length ranged from 12 bp to 47 bp. Twenty-four (63.1%) microsatellites were perfect, two (5.3%) were imperfect and 12 (31.6%) were compound repeats. From those, 16 clones were chosen to design the primers, since they had more than 10 repeats. Microsatellite sequences formed by less than 10 repeats are considered to be less polymorphic, and thus not very informative.

Seven clones contained AG/TC repeats, three contained AC/TG repeats, five contained AT/TA repeats, and one contained a poly A repeat [(A)_35_GG(A)_9_]. Sixty-three percent of the selected clones (10/16) had complementary sequences to the oligonucleotides used in the enrichment procedure. However, the other 37% had different repeats (AT and A) that were not totally complementary to the probes used. The selection of AT sequences using AC and AG oligonucleotides were not reported in other studies where libraries were enriched for these two types of sequences [25-27]. In the previous studies the hybridization between the probes and single stranded clones were performed at temperatures superior to 50°C, thus under very stringent conditions, reducing the possibility of selection of clones due to mismatches. In this study, the enrichment was performed at room temperature (around 25°C). Some sequences did not contain repeated sequence indicating that mismatches have happened. However, the frequency of AT in this group was high. This high percentage could be due to the probes used (AC_15 _and AG_15_), which could have had up to 50% of their sequences complementary to AT/TA regions. Taking into account only the adenines in these probes, since adenines would pair to the timidines of target or part of the target sequence, temperatures above 35°C would be necessary to break the nitrogen bonds, since 35°C is the melting point of an oligonucleotide formed by 15 adenines. The forementioned temperature is 10°C higher than the room temperature, allowing a more stable association between the probe and the adenine-rich target (AT and poliA) than in adenine-poor targets, increasing the frequency of these motifs in the group of selected sequences due to the mismatch.

Primers were designed and synthesized to 13 of the 16 sequences selected. This set of primers and a primer pair developed by Hopkins and colleagues [15] were used to amplify microsatellite loci in *A. hypogaea *and in wild diploid species of eight sections of genus *Arachis*.

The 14 primer pairs allowed the detection of 18 putative loci in *A. hypogaea *(Table 1). Thus, a number of primer pairs amplified loci in both genomes of *A. hypogaea*. Four primer pairs (Ah7, Ah21, Ah30 and Ah282) amplified two putative loci in *A. hypogaea *and one locus in *A. duranensis *(genome A) and *A. ipaënsis *(genome B) and the other ten pairs allowed the amplification of a single putative locus that was amplified in *A. hypogaea *and in *A. duranensis *or *A. ipaënsis *(data not shown). Thus, the primers pairs fall into three groups based on the amplification events observed in *A. hypogaea *and in *A. ipaënsis *and *A. duranensis*: 1) those allowing the amplification in *A. hypogaea *and *A. duranensis *and detect a putative locus in the A genome, 2) those allowing the amplification of a putative locus in *A. hypogaea *and *A. ipaënsis *and detect a locus in the B genome, and 3) those allowing the amplification in *A. hypogaea*, *A. duranensis *and *A. ipaënsis *and detect putative loci in both genomes.

The level of polymorphism varied greatly among the polymorphic loci. Ah51 allowed the amplification of seven alleles and the PIC was 0.79, whereas the least polymorphic primer pair Ah282 amplified only two alleles and presented PIC = 0.11. Primers Ah51 flank a region that comprises the largest number of repeats (34) and the motif was formed by two nucleotides (A and G) whereas Ah282 flanked a region containing two microsatellites, each composed of six trinucleotide repeats. Hopkins and colleagues [15] and He and colleagues [28] observed that some loci, despite their long repeats (20–40), were invariant among the cultivated accessions tested. The difference observed in the studies cited above may have been due to the following: 1 – distinct number of loci were analyzed in these studies; 2 – distinct sets of *A. hypogaea *accessions were used. Moreover, the invariant microsatellites may be located in genes, what make them less variable despite their long repeats.

Overall, the mean percentage of polymorphic loci was 33%, the mean number of alleles per primer pair within the accessions of *A. hypogaea *was 4.02, and the PIC was 0.48. In this study, the percentage of polymorphic microsatellite loci was lower than those found in other studies where microsatellite markers were used to evaluate genetic variability within *A. hypogaea*. Ferguson and colleagues [16] studying a set of 24 accessions of *A*. *hypogaea *from 7 countries from different continents found 57.3% of polymorphic microsatellite loci. He and colleagues [28] found that 34% of the microsatellite primer pairs showed polymorphism in a sample that comprised *A. hypogaea *accessions from eight Latin America countries. Despite the lower percentage of microsatellite loci found in this study, it was higher than the percentage of polymorphic loci in *A. hypogaea *observed using RAPD [6.6% (29)], and AFLP [6.7% (6); 6.4 %, (30)]. Besides the large percentage of polymorphic loci, Hopkins and colleagues [15] observed that the amount of useful information obtained per polymorphic microsatellite locus was quite high. For instance, in this study, the mean number of alleles was 4.02, and several primer pairs were highly informative, such as primer pair Ah51, which allowed the amplification of seven different fragments.

PCR products were obtained for most of the wild species analyzed (Table 2). In general, fragments close to the size of the fragment expected for *A. hypogaea *were detected. The transferability of the markers was variable, ranging from 54% for the locus Ah6–125 to 100% for Ah30. The level of polymorphism also varied among loci, ranging from 25 alleles in Ah30 and Ah126 to 15 alleles in Ah11 and Ah20.

**Figure 1:**
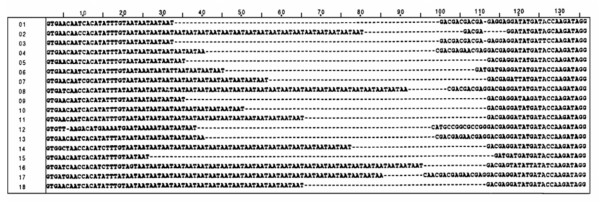
Alignment of nucleotide sequences of 18 *Arachis *species amplified using primer pair Ah11. Species analyzed: *A. valida *(1), *A. triseminata *(2), *A. subcoreacea *(3), *A. cardenasii *(4), *A. guaranitica *(5), *A. batizocoi *(6), *A. repens *(7), *A. duranensis *(8), *A. paraguariensis *(9), *A. burkartii *(10), *A. ipaënsis *(11), *A. kuhlmannii *(12), *A. kempff-mercadoi *(13), *A. magna *(14), *A. hoehnei *(15), *A. decora *(16), *A. macedoi *(17) and *A. hypogaea *(18). The sequences of all species comprised microsatellites, but the number of repeats varied a lot among them.

The annealing temperatures used to amplify microsatellite loci in wild *Arachis *species ranged from 10°C below the melting temperature (Tm) of a given pair of primers to the melting temperature of the primer. The necessity of lower annealing temperatures for some pairs of primer suggested that some microsatellite flanking regions were more conserved than others in the *Arachis *species analyzed. The data also suggested that changes in the flanking regions most probably resulted from point mutations and small deletions and insertions, since if major rearrangements were responsible for causing the changes, they would probably have resulted in no amplification due to the interruption or deletion of primer-annealing sites. Point mutations and small rearrangements (deletions and/or insertions) were detected in the flanking regions of the Ah11 locus of some species analyzed (Figure 1). For instance, a sequence of five bases (positions 112 to 116) was absent from *A. triseminata*.

The cross-transferability of *A. hypogaea *markers to species of section *Arachis *was very high, ranging from 60% for Ah20 to 100% for Ah30. A similar level of microsatellite marker transferability was observed from *Triticum aestivum *L. to its ancestral diploid species [24]. Section *Arachis *comprises species with genomes (AA and BB) similar to those found in the cultivated peanut (AABB) showing agronomical value characteristics, which are introgressed into cultivated peanut mainly by means of crosses with synthetic amphidiploids resultant from crosses between A and B genome species. The resulting F_1 _has to be backcrossed many times to get an off-spring that has the introgressed characteristic and most of the recurrent parental genome. A genetic map constructed using wild diploid species would be useful to guide the introgression of genes from wild species to *A. hypogaea*. A map would allow the discovery of markers linked to gene(s) or chromosome regions that are responsible or involved in the expressions of introgressed characteristic. Similarly, it would allow the selection of markers distributed all over both genomes of *A. hypogaea*, helping the selection of plants showing largest percentages of the recurrent parental genome. This approach would be the most efficient way to integrate molecular markers into breeding programs of cultivated peanut, since genetic polymorphism in *A. hypogaea *is very low [4,15,31] and insufficient to construct a genetic map.

**Figure 2:**
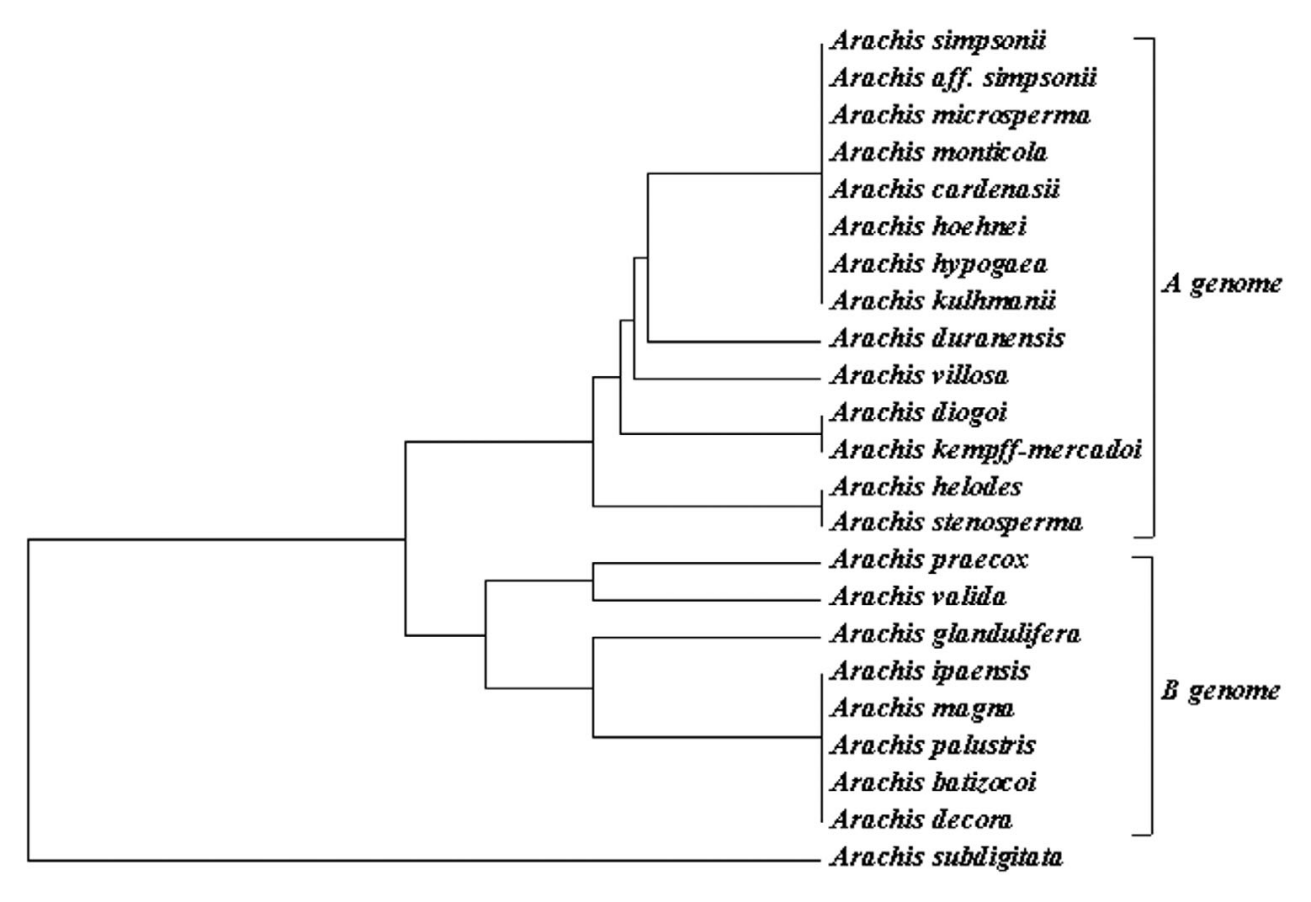
Phenogram showing the relation among *Arachis *species based on amplification events obtained using eight primer pairs and 22 *Arachis *species. The polymorphism was not enough to characterize most species, but they were grouped according the type of their genomes (A, B and D).

Figure 2 shows the relationships among species of *Arachis *section based on amplification events observed using eight pairs of microsatellite loci from *A. hypogaea*. Two groups were identified. The first consisting of *A. hypogaea*, *A. monticola *and all analyzed genome Aspecies, the second contained the species classified as genome B and *A. glandulifera *Stalker, classified as genome D [32]. The division into two main groups was based essentially on the absence of amplification of two loci (Ah20 and Ah6–125) in genome B species. Despite the few loci analyzed (8), the groups defined by the dendrogram agreed with previous studies that classified species of the *Arachis *section into genomes A, B and D. In this study A genomes species were placed closer to *A. hypogaea *than the B genomes. Tallury and colleages [33] using AFLPs found *A ipaënsis *and *A. williamsii*, both B genome species, closer to *A. hypogaea *than A genome species. This difference on affinities of A and B genome species with *A. hypogaea *may be due to the type and number of markers used. The data also agreed with the close relationship between *A. glandulifera *and B genome species [33]. These findings suggested that flanking regions contain useful phylogenetic information.

PCR products were also obtained for species from sections *Caulorrhizae, Erectoides, Extranervosae, Procumbentes, Rhizomatosae, Trierectoides *and *Triseminatae *(Table 3). Five primer pairs, namely Ah2, Ah11, Ah19, Ah30 and Ah126, from the eight primers (62.5%) tested resulted in amplifications from all sections. The pair Ah6–125 (12.5%) produced amplification in six sections, and Ah20 and Ah21 in five sections (25%). Taking into account that 33% of the primers pairs allowed the detection of polymorphism among accessions of *A. hypogaea*, this set of primers will probably show polymorphism in wild *Arachis *species, so they could be analyzed using microsatellite loci with no costs to primer development. Cross-transferability of *Arachis *microsatellite markers was also observed in other studies. Hopkins and colleagues [15] using *A. hypogaea *microsatellite primers observed cross-amplification in *A. monticola*, *A. ipaënsis *and *A. duranensis*, species that are closely related to *A. hypogaea*. Moretzsohn and colleagues [17] also using *A. hypogaea *microsatellite primers observed up to 76% of transferability to species of *Arachis *section and up to 45% to species of the other eight section of genus *Arachis*. Moretzsohn and colleagues [34] observed cross amplification and detected polymorphism between *A. duranensis *(A genome) and *A. stenosperma *(A genome) using a large number of pair of primers developed for different *Arachis *species.

The existence of repeated sequences in microsatellite primer-amplified fragments for locus Ah11 of *A. hypogaea *and DNA of 18 species (*A. batizocoi A. burkartii, A. cardenasii, A. decora*, *A. duranensis, A. guaranitica, A. hoehnei, A. hypogaea, A. ipaënsis, A. kempff-mercadoi, A. kuhlmannii, A. macedoi, A. magna, A. paraguariensis, A. repens, A. subcoriacea, A. triseminata *and *A. valida*) was confirmed by sequencing. The sequences of the fragments of each species analyzed are shown in Figure 1. All species showed repeated sequences similar to those found in Ah11 locus of the cultivated peanut, regardless of the section to which the species belonged. These sequences differed from each other only in the number of repeated motifs. Thus, primers for *A. hypogaea *were able to amplify microsatellites in other *Arachis *species.

**Figure 3:**
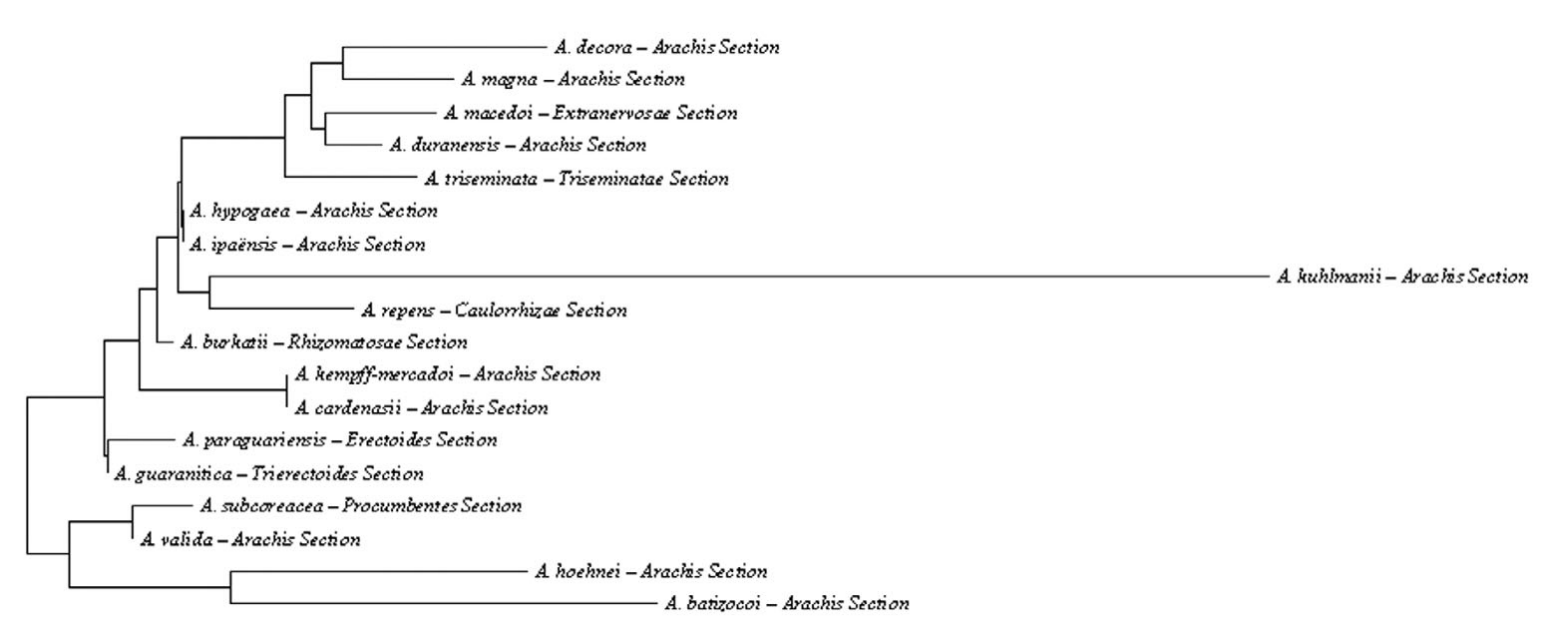
Relationships among *A. hypogaea *and 17 *Arachis *(*A. valida, A. triseminata, A. subcoreacea, A. cardenasii, A. guaranitica, A. batizocoi, A. repens, A. duranensis, A. paraguariensis, A. burkartii, A. ipaënsis, A. kuhlmanni, A. kempff-mercadoi, A. magna, A. hoehnei, A. decora, A. macedoi*) species based on polymorphism found in the sequences amplified using primer pair Ah11.

A neighbor-joining tree constructed based on a small part of the flanking regions and on the repeated sequences of the Ah11 locus in 18 species is shown in Figure 3. The species of the different sections of *Arachis *were scattered throughout the tree and some were located close to species from other sections. The majority of the variation among species reflected differences in the number of motifs among the species and not in the flanking regions. These results suggested that there was no correlation between the number of repeated sequences and the taxonomic relationship among these species, and that the level of information contained in a microsatellite locus did not necessarily positively correlate to the degree of relatedness to *A. hypogaea*. For instance, *A. hoehnei* and *A. cardenasii*, both from section *Arachis*, had shorter microsatellites than *A. repens* (Section *Caulorrhizae*) and *A. triseminata* (section *Triseminatae*).  A larger number of plants from each species would need to be analyzed in order to test this hypothesis because microsatellites are highly polymorphic and the accessions of the species analyzed may have been extreme in the range of variation found at each analyzed locus.

An analysis of the cross-transferability of microsatellite loci in *Vitaceae *showed that microsatellite repeats were present in most of the species examined and that flanking sequences were conserved and could be used to examine evolutionary relationships [35]. The potential usefulness of flanking regions to assess taxonomic relationship in *Arachis *was not approached in this study. However, our results indicate that these regions could be useful for establishing genetic relationship in *Arachis*, since the relationship established based on amplification events (Figure 2), which depend on the conservation of the flanking regions, agreed with the division of the species of *Arachis *section into genomes A and B.

Some species had more than one fragment amplified using some primer pairs, including accessions of the diploid species *A. simpsonii*, *A. aff. cardenasii*, *A. linearifolia*, *A. hermannii*, and *A. pseudovillosa*, which showed more than one fragment using Ah21 (Table 4). These results suggested that the accessions of the above species were heterozygous. *Arachis *species were expected to be homozygous since they are considered to be autogamous simply by analogy to cultivated peanut [36]. In addition, these species are diploid, a fact that excludes the possibility of existence of two homozygous loci with different alleles, as observed for Ah21 and Ah30 in *A. hypogaea*, which is an allotetraploid (Table 3). The data suggested that cross-pollination happens in some *Arachis *species. Evidences of cross-pollination in *A. duranensis *were found when different accessions were analyzed using RFLP [37]. The extensive polymorphism detected within accessions of *A. cardenasii *using cDNA and seed storage proteins probes [5,38,39] has also been suggested to be related to high frequency of cross-pollination. As polymorphic codominant markers, microsatellites are useful tools to analyze the mating system of wild species of *Arachis*.

## Conclusion

These results show that microsatellite primer pairs of *A. hypogaea *have multiple uses. A higher level of variation among *A. hypogaea *accessions is detected using microsatellite markers in comparison to other markers, such as RFLP, RAPD and AFLP. The microsatellite primer pairs of *A. hypogaea *showed high transferability rate to other species of the genus. These primer pairs are useful tools to evaluate the genetic variability and to assess the mating system among *Arachis *species.

## Methods

### Plant material

Sixteen accessions of *A. hypogaea *and 38 accessions of species from eight of the nine sections of the genus *Arachis *were analyzed (Table 3). The samples were obtained from *Arachis *Germplasm Bank (EMBRAPA Recursos Genéticos e Biotecnologia – Brasília, DF, Brazil).

### DNA extraction

DNA was extracted using the procedure of Grattapaglia and Sederoff [40]. The quality of the DNA was checked on 1% agarose gels and the DNA concentrations were estimated spectrophotometrically (Genesys 5 – Spectronic Instruments).

### Library construction and primer design

Nine micrograms of genomic DNA from *A. hypogaea *were digested using 1.35 μl of *Hae*III (10 U/μl), 2 μl of *Alu*I (10 U/μl) and 1 μl of *Rsa*I (10 U/μl) (New England Biolabs). The reaction products were electrophoresed on 1% low melting point agarose gels and fragments 200–600 bp in size were excised from the gel, extracted with phenol/chloroform, and ligated into pBluescript (Stratagene). The ligated clones were used to transform *Escherichia coli *XL1-blue MRF' (Stratagene). The library was amplified overnight at 30°C with shaking (300 rpm) and the plasmids then isolated by phenol extraction. The library was enriched for AC and AG repeats using a GeneTrapper^® ^cDNA positive selection system (Invitrogen). The selected clones were used to transform XL1-blue MRF' bacterial cells. The white colonies were grown overnight in LB-liquid medium supplemented ampicillin (100 μg/ml). The plasmids were extracted using a Concert^® ^rapid plasmid purification system (Invitrogen). Sequencing was done using T3 and T7 primers. The sequencing reaction mixture consisted of 2 μl of plasmid DNA, 2 μl of Big Dye terminator, 8 pmol of primer and water to a final volume of 10 μl. Sequencing was carried out in an ABI Prism 377 sequencer (Applied Biosystems). The primers were designed using Primer3 software [41].

### PCR

Fourteen primer pairs were used for PCR amplification, being 13 designed using the sequences selected in the above step and one reported by Hopkins and colleagues [15] (Table 4). Each amplification reaction contained 1 U of *Taq *DNA polymerase (Invitrogen), 1 × amplification buffer (200 mM Tris pH 8.4, 500 mM KCl), 200 μM of each dNTP, 1.5–2.5 mM MgCl_2 _(Table 2), 0.2 μM of each primer, 15 ng genomic DNA, and water to a final volume of 17 μl. The following thermocycling conditions were used: 1 cycle: 94°C for 5 min, 35 cycles: 94°C for 30 s, × °C for 45 s and 72°C for 1 min, and a final cycle: 72°C for 10 min. The annealing temperatures (X) and MgCl_2 _concentrations were optimized for each pair of primers to allow amplification in the wild species. The amplifications were done in a PTC100 thermocycler (MJ Research).

### Electrophoresis

The sequence variation in *A. hypogaea *and two wild diploid species (*A*. *duranensis *and *A. ipaënsis*) was analyzed using 4% denaturating polyacrylamide gels (19:1 acrylamide/bisacrylamide, 7 M urea) that were silver stained. The sizes of the fragments were estimated based on a 10 bp ladder (Invitrogen).

The PCR products obtained using DNA from wild species were electrophoresed on 3% metaphor (FMC Bioproducts) agarose gels for 3 h at 120 V. The agarose gels were stained with ethidium bromide and PCR products viewed under UV light. The size of fragments was estimated based on a 100 bp ladder (GE).

### Analysis of variation in *A. hypogaea*

The allelic and genotypic frequencies were calculated for the samples analyzed. The genetic variability of the sample as a whole was estimated based on the number of alleles per locus (total number of alleles/number of loci), the percentage of polymorphic loci (number of polymorphic loci/total number of loci analyzed) and Polymorphism Information Content (PIC = 1 - ∑i=1Pi2−∑i=1∑j=i+1Pi2Pj2
 MathType@MTEF@5@5@+=feaafiart1ev1aaatCvAUfKttLearuWrP9MDH5MBPbIqV92AaeXatLxBI9gBaebbnrfifHhDYfgasaacH8akY=wiFfYdH8Gipec8Eeeu0xXdbba9frFj0=OqFfea0dXdd9vqai=hGuQ8kuc9pgc9s8qqaq=dirpe0xb9q8qiLsFr0=vr0=vr0dc8meaabaqaciaacaGaaeqabaqabeGadaaakeaadaaeqbqaaiabbcfaqjabbMgaPnaaCaaaleqabaGaeGOmaidaaaqaaiabbMgaPjabg2da9iabigdaXaqab0GaeyyeIuoakiabgkHiTmaaqafabaWaaabuaeaacqqGqbaucqqGPbqAdaahaaWcbeqaaiabikdaYaaakiabbcfaqjabbQgaQnaaCaaaleqabaGaeGOmaidaaaqaaiabbQgaQjabg2da9iabbMgaPjabgUcaRiabigdaXaqab0GaeyyeIuoaaSqaaiabbMgaPjabg2da9iabigdaXaqab0GaeyyeIuoaaaa@4B09@).

### Analysis of the locus cross-species transferability

The cross-species transferability of eight loci was evaluated using 37 accessions of 37 species from eight sections of the genus *Arachis*. The percentage of transferability was calculated for each locus for section *Arachis *(22 species) and for the whole sample (37 species) as the number of species in which the expected fragment was detected/the total number of species analyzed. A binary matrix based on the amplification events for section *Arachis *alone was prepared based on the data in Table 4. In this matrix, 1 indicated amplification and 0, no amplification. A genetic distance matrix was calculated using the Nei and Li distance [42] and a dendrogram was constructed using the UPGMA method (unweighted pair group method with arithmetic mean) [43].

### Sequencing of PCR products and sequence analysis

The PCR products obtained using the pair of primers for locus Ah11 and DNA of 18 species (*A. batizocoi A. burkartii, A. cardenasii, A. decora*, *A. duranensis, A. guaranitica, A. hoehnei, A. hypogaea, A. ipaënsis, A. kempff-mercadoi, A. kuhlmannii, A. macedoi, A. magna, A. paraguariensis, A. repens, A. subcoriacea, A. triseminata *and *A. valida*) were purified using the Concert^® ^Rapid PCR purification system (Invitrogen). The sequencing reaction mixture had a total volume of 10 μl: 2 μl of purified PCR product, 2 μl of Big Dye Terminator, 6 pmol of one primer, and 5.4 μl of water. The sequencing cycle consisted of 25 cycles of 96°C for 45 s, 55°C for 55 s, and 60°C for 4 min. The reactions were run in a PTC 100 cycler (MJ Research) followed by sequencing in an ABI Prism 377 sequencer. The sequences were edited using the Sequencer program (version 3.1) (GeneCodes). Sequence alignment and a neighbor-joining tree were obtained using Clustal X (version 1.8) [44].

## Authors' contributions

AAH, AVGB and DAP have made substantial contributions in the acquisition, analysis and interpretation of data; MAG and CRL have made contributions to conception, design and interpretation of data. All authors have been involved in revising the manuscript critically and approved the final version.

## Figures and Tables

**Table 1:** Data of each of the 18 putative microsatellite loci of *A. hypogaea*.

**Locus**		**Range (bp)**	**Allele Frequencies**							**PIC**
			**1**	**2**	**3**	**4**	**5**	**6**	**7**	
Ah2	199	199	1.00	-	-	-	-	-	-	0.00
Ah3	202	220–188	0.03	0.12	0.56	0.18	0.06	0.09	-	0.66
Ah7.1	102	104	1.00	-	-	-	-	-	-	0.00
Ah7.2		102	1.00	-	-	-	-	-	-	0.00
Ah11.1	176	180–175	0.41	0.18	0.41	-	-	-	-	0.65
Ah19	197	175	1.00	-	-	-	-	-	-	0.00
Ah20	197	199–197	0.93	0.07	-	-	-	-	-	0.13
Ah21.1	109	114	1.00	-	-	-	-	-	-	0.00
Ah21.2		111	1.00	-	-	-	-	-	-	0.00
Ah23	183	165	1.00	-	-	-	-	-	-	0.00
Ah26	182	190–178	0.24	0.12	0.35	0.24	0.06	-	-	0.77
Ah30.1	123	127	1.00	-	-	-	-	-	-	0.00
Ah30.2		124	1.00	-	-	-	-	-	-	0.00
Ah51	154	152–124	0.17	0.07	0.40	0.17	0.07	0.07	0.07	0.79
Ah6–125	180	159	1.00	-	-	-	-	-	-	0.00
Ah126	187	200	1.00	-	-	-	-	-	-	0.00
Ah282.1	203	202–196	0.06	0.94	-	-	-	-	-	0.11
Ah282.2		182	1.00	-	-	-	-	-	-	0.00

**Table 2:** Sizes (bp) of fragments amplified using eight *A. hypogaea *microsatellite primer pairs and DNAs of 37 wild *Arachis *species.

	**Ah2**	**Ah11**	**Ah19**	**Ah20**	**Ah21**	**Ah30**	**Ah6–125**	**Ah126**
**Section *Arachis***								
*A. batizocoi*	286	146	151	--	135	139	--	210
	330							
*A. cardenasii*	248	155	143	203	139	139	180	191
	200		149		141			
*A. decora*	200	194	144	--	136	135	--	185
								203
*A. aff. diogoi*	279	--	143	205	137	126	197	213
	242		149		140		180	
	205							
*A. duranensis*	181	194	143	--	134	140	191	187
	205		147					
*A. glandulifera*	242	--	149	--	140	128	--	216
*A. helodes*	242	150	143	208	137	124	174	--
	205	155	147			157		
*A. hoehnei*	215	150	143	196	143	142	186	224
			147					203
*A. ipaënsis*	231	161	151	--	132	130	--	203
*A. kempff-mercadoi*	286	--	143	200	142	140	186	213
	196		147					
*A. kuhlmannii*	242	155	142	196	138	137	171	200
	200		147					
*A. magna*	231	173	151	--	136	139	--	200
*A. microsperma*	286	130	145	189	134	144	178	213
	205		149					
*A. monticola*	231	157	151	196	133	126	182	203
	196	169						
*A. palustris*	200	150	151	--	135	130	--	210
*A. praecox*	--	155	143	--	141	123	--	191
			144					198
*A. simpsonii*	200	155	145	203	137	144	180	205
	341				140			
*A. aff. simpsonii*	196	153	143	205	140	133	204	193
	248		149				177	
*A. stenosperma*	293	145	142	193	144	130	182	--
	215	150	149					
*A. subdigitata*	--		--	183	--	123	166	196
							221	
*A. valida*	--	161	143	--	135	139	184	198
*A. villosa*	--	155	143	187	134	144	178	203
			149					
**Section *Caulorrhizae***								
*A. pintoi*	205	161	143	183	147	128	208	205
			151					
			166					
*A. repens*	205	--	162	--	140	130	--	239
			170					
**Section *Erectoides***								
*A. paraguariensis*	200	--	162	167	141	119	208	230
	187			173				
*A. hermannii*	196	--	--	179	139	130	--	219
					145			
*A. major*	200	153	151	187	144	121	170	230
			162					
**Section *Extranervosae***								
*A. burchellii*	197	188	142	--	165	132	--	200
						153		
						284		
*A. pietrarellii*	300	--	141	--	--	129	--	209
						155		
*A. prostata*	189	--	139	--	133	121	--	194
					136	153		205
*A. macedoi*	--	--	142	182	--	122	180	214
			155					
*A. villosulicarpa*	--	160	144	--	152	124	--	188
						149		
**Section *Rhizomatosae***								
*A. burkartii*	--	139	178	175	136	125	--	196
			241					232
*A. glabrata*	195	146	158	169	137	125	--	180
			161					192
								205
*A. pseudovillosa*	203	146	158	169	135	122	--	186
			164		138			209
								226
								239
**Section *Trierectoides***								
*A. guaranitica*	203	189	158	--	137	117	182	196
			164					
**Section *Triseminatae***								
*A. triseminata*	187	--	164	--	143	180	--	224
	203							

**Table 3:** Species evaluated using *A. hypogaea *microsatellite primers.

**Section**	**Species**	**Ploidy**	**Genome**	**Collector's numbers**	**State and country**
*Arachis*	*Arachis batizocoi*	2n = 20	B	K9484	Bolivia
	*Arachis aff. cardenasii*	2n = 20	A	V13721	MT, Brazil
	*Arachis decora*	2n = 18	Unknown	V13290	GO, Brazil
	*Arachis linearifolia*	2n = 20	Unknown	V9401	MT, Brazil
	*Arachis duranensis*	2n = 20	A	V14167	Argentina
	*Arachis glandulifera*	2n = 20	D	V13738	MT, Brazil
	*Arachis helodes*	2n = 20	A	V6325	MT, Brazil
	*Arachis hoehnei*	2n = 20	Unknown	V9140	MS, Brazil
	*Arachis hypogaea*	2n = 40	AB	W725	GO, Brazil
		2n = 40	AB	AsW433	RO, Brazil
		2n = 40	AB	URY85183	Rivera, Uruguay
		2n = 40	AB	Pd3147	RS, Brazil
		2n = 40	AB	V12577	MT, Brazil
		2n = 40	AB	URY85273	Rivera, Uruguay
		2n = 40	AB	URY85209	Rivera, Uruguay
		2n = 40	AB	V12577-1	MS, Brazil
		2n = 40	AB	Mf1640	Ecuador
		2n = 40	AB	Mf1670	Ecuador
		2n = 40	AB	Mf1600	Ecuador
		2n = 40	AB	V12548	MT, Brazil
		2n = 40	AB	V10522	SC, Brazil
		2n = 40	AB	Tatu	SP, Brazil
		2n = 40	AB	Tatu ST	SP, Brazil
		2n = 40	AB	166	Not available
	*Arachis ipaënsis*	2n = 20	B	K30076	Bolívia
	*Arachis kempff-mercadoi*	2n = 20	A	V13530	MS, Brazil
	*Arachis kuhlmannii*	2n = 20	A	V6344	MT, Brazil
	*Arachis magna*	2n = 20	B	K30097	Santa Cruz, Bolívia
	*Arachis microsperma*	2n = 20	A	Sv3837	Paraguay
	*Arachis monticola*	2n = 40	AB	V14165	Argentina
	*Arachis palustris*	2n = 18	B	V13023	TO, Brazil
	*Arachis praecox*	2n = 18	B	V6416	MT, Brazil
	*Arachis simpsonii*	2n = 20	A	V13728	Bolívia
	*Arachis aff. simpsonii*	2n = 20	A	V13746	MT, Brazil
	*Arachis stenosperma*	2n = 20	A	V10309	MT, Brazil
	*Arachis subdigitata*	2n = 20	A	V13589	Not available
	*Arachis valida*	2n = 20	B	V14041	Not available
	*Arachis villosa*	2n = 20	A	V9923	Not available
*Caulorrhizae*	*Arachis pintoi*	2n = 20	C	V13356	BA, Brazil
	*Arachis repens*	2n = 20	C	V5868	RS, Brazil
*Erectoides*	*Arachis paraguariensis*	2n = 20	E	V13546	MS, Brazil
	*Arachis hermannii*	2n = 20	E	V10390	MS, Brazil
	*Arachis major*	2n = 20	E	V7644	MT, Brazil
*Extranervosae*	*Arachis burchellii*	2n = 20	EX	S3766	TO, Brazil
	*Arachis pietrarellii*	2n = 20	EX	V12085	MT, Brazil
	*Arachis prostrata*	2n = 20	EX	W722	GO, Brazil
	*Arachis macedoi*	2n = 20	EX	V9912	Not available
	*Arachis villosulicarpa*	2n = 20	EX	V8816	MT, Brazil
*Procumbentes*	*Arachis subcoriacea*	2n = 20	E	V8943	MT, Brazil
*Rhizomatosae*	*Arachis burkartii*	2n = 20	R	Ff1122	RS, Brazil
	*Arachis glabrata*	2n = 40	R	V7300	MG, Brazil
	*Arachis pseudovillosa*	2n = 20	R	V7695	MS, Brazil
*Trierectoides*	*Arachis guaranitica*	2n = 20	E	V13600	MS, Brazil
*Triseminatae*	*Arachis triseminata*	2n = 20	T	W 195	BA, Brazil

Abbreviations: As – Scariot, Ff – Ferreira, K - Krapovickas, Sv - Silva, V - Valls, W- Werneck

**Table 4:** PCR primer pairs used to amplify microsatellites in wild species of *Arachis*

**Locus**	**Motifs**	**Primer sequences (5'-3')**		**Expected size (bp)**	**Annealing temperature (°C)**	**MgCl_**2 **_mM**
					
		**Forward**	**Reverse**			
Ah2	(AC)_10_	TTACACGGTAGCCCATTTCC	CCAAACCACAATTCAGTAGCAA	199	55	2.5
Ah3	(GA)_15_.(AG)_7_.(GT)_7_.(GA)_7_	TCGGAGAACAAGCACACATC	TTGCGCTCTTTCTCACACTC	202	55	1.5
Ah7	(TG)_8_	CAGAGTCTGTGATTTGTGCACTG	ACAGAGTCGGCCGTCAAGTA	102	55	1.5
Ah11	(TTA)_15_	AAATAATGGCATACTTGTGAACAATC	TTCCACCAAGGCAAGACTATG	176	55	2.5
Ah19	(TA)_18_	CCCTCGAAGGTGGAATTCAT	CGGGGATTGTTCGAGTTTG	197	55	2.5
Ah20	(TG)_10_	TGCATGTCTCTTGTAACTTAATACACT	TTCATGTCAATGATGTTTCCAA	197	55	2.5
Ah21	(GAA)_9_	CTTGGAGTGGAGGGATGAAA	CTCACTCACTCGCACCTAACC	109	55	1.5
Ah23	(AT)_19_	GAAGGTGGAATTCATTCTCAAAA	TTCGAGTTTGAACAACTGACG	183	55	2.0
Ah26	(CT)_14_	GAAAATGATGCCATAAAGCGTA	AGTGTAACACCCCGTTAGCC	182	55	2.0
Ah30	(GA)_9_	TGCTCTTCTTTTCCTTTTCAC	AACGGCCAAAACTGAAATTA	123	45	2.0
Ah51	(AG)_34_	CCTCTTCACAAGAGTGGACTATGA	CCCCCTCCTTTTGTTCTCTC	154	55	2.0
Ah6–125*	(GAA)_13_	TCGTGTTCCCGATTGTCC	GCTTTGAACATGAACATGCC	180	55	2.0
Ah126	(GA)_8_..(GA)_9_	CCCTGCCACTCTCACTCACT	CGTACAAGTCAGGGGGTGAC	187	60	1.5
Ah282	(CCA)_6_..(AAG)_6_	GCCAAACACACCACATTTCA	GGCTCCAATCCCAAACACTA	203	55	2.5

* Reference: Hopkins and colleagues [15].
